# Protecting Life While Preserving Liberty: Ethical Recommendations for Suicide Prevention With Artificial Intelligence

**DOI:** 10.3389/fpsyt.2018.00650

**Published:** 2018-12-03

**Authors:** Lindsey C. McKernan, Ellen W. Clayton, Colin G. Walsh

**Affiliations:** ^1^Department of Psychiatry and Behavioral Sciences, Vanderbilt University Medical Center, Nashville, TN, United States; ^2^Department of Physical Medicine and Rehabilitation, Vanderbilt University Medical Center, Nashville, TN, United States; ^3^Center for Biomedical Ethics and Society, Vanderbilt University Medical Center, Nashville, TN, United States; ^4^Law School, Vanderbilt University, Nashville, TN, United States; ^5^Department of Biomedical Informatics, Vanderbilt University Medical Center, Nashville, TN, United States; ^6^Department of Medicine, Vanderbilt University Medical Center, Nashville, TN, United States

**Keywords:** artificial intelligence, suicide, ethics, code of ethics, machine learning

## Abstract

In the United States, suicide increased by 24% in the past 20 years, and suicide risk identification at point-of-care remains a cornerstone of the effort to curb this epidemic (1). As risk identification is difficult because of symptom under-reporting, timing, or lack of screening, healthcare systems rely increasingly on risk scoring and now artificial intelligence (AI) to assess risk. AI remains the science of solving problems and accomplishing tasks, through automated or computational means, that normally require human intelligence. This science is decades-old and includes traditional predictive statistics and machine learning. Only in the last few years has it been applied rigorously in suicide risk prediction and prevention. Applying AI in this context raises significant ethical concern, particularly in balancing beneficence and respecting personal autonomy. To navigate the ethical issues raised by suicide risk prediction, we provide recommendations in three areas—communication, consent, and controls—for both providers and researchers (2).

In the United States, suicide increased by 24% in the past 20 years, and suicide risk identification at point-of-care remains a cornerstone of the effort to curb this epidemic (1). As risk identification is difficult because of symptom under-reporting, timing, or lack of screening, healthcare systems rely increasingly on risk scoring and now applied artificial intelligence (AI) to assess risk. AI remains the science of solving problems and accomplishing tasks, through automated or computational means, that normally require human intelligence. Applied AI specifically focuses on enabling high-functioning systems to replicate human intelligence for a dedicated purpose, such as risk prediction (3). This science is decades-old and includes traditional predictive statistics and machine learning. Only in the last few years has it been applied rigorously in suicide risk prediction and prevention. These approaches raise significant ethical challenges. AI has the potential to prompt recommendations for suicidal patients including monitoring, evaluation, and intervention. At the most extreme, involuntary hospitalization for those at highest risk brings the goal of preserving life directly in conflict with the immediate liberty interests of the patient. To navigate the ethical issues raised by suicide risk prediction, we provide recommendations in three areas—communication, consent, and controls—for both providers and researchers (2).

Predicting suicide risk remains a key challenge in suicide prevention, with risk misclassification having serious consequences–both “false negatives” who go on to self-harm without being identified and “false positives” who are monitored, screened, or treated unnecessarily, although the frequency and severity of these harms is not well-known.

Until now, suicide screening occurred during interpersonal interaction using clinician judgment. The use of AI has advanced our ability to predict suicide risk across all ages and in diverse populations (4–7). For example, recent models predict suicide attempt risk with excellent accuracy (>90%) and good precision (>80%). Novel applications of predictive models can both reinforce known risk factors that might be missed and identify potentially novel risk factors at the same time. For instance, insomnia has been demonstrated as an important risk factor of suicidality and in preceding the use of violent methods in particular (8–10). Our research in both adults and adolescents identified surrogates for sleep disorder with the use of natural language processing in the context of AI (6, 11). We noted documentation of melatonin use even in the absence of documented sleep disorder as a strong risk factor of suicide attempts. Similar surrogates of psychosis, depression, and more well-known risk factors have been identified in this way.

In addition to reinforcing known research, AI might identify novel risk and protective factors in those at increased risk of suicide. In a large cohort of patients with fibromyalgia, polysomatic symptoms such as frequent clinical encounters for “weakness,” “fatigue,” and “dizziness” correlated strongly with increased suicidality. Further analyses showed that high rates of outpatient engagement were significant protective factors that also might suggest a prevention strategy. All of these patterns were identified through validated AI, and none had been shown prior to our knowledge (12).

Despite the promise of AI in this domain, caveats exist. The high performance metrics above were reported in case-control studies and might not replicate to prospective cohorts. Risk models of rare events like suicide remain prone to low precision, which means many false positives must be screened to identify one true positive (13). Patterns in known and novel risk factors are correlative and not causative without significant and dedicated further study. We do not know how patients and providers will react to this new technology. We do not have broadly-accepted practice standards or guidelines for implementation (14). Institutional policies may not address the particular challenges of using AI in this way. Thus, we risk adopting AI into clinical practice at ethical cost–not just financial cost.

Ethical challenges also pervade suicide research. For example, involuntary commitment threatens the balance between beneficence (preventing suicide) and autonomy (respecting patient choice). Multiple accurate and large-scale prediction models have been recently published in both civilian and military settings (4–7) The emphasis remains on novel prediction, however, and not on the literacy, numeracy, and education necessary to integrate AI technologies (and medical innovation in general) effectively and ethically into practice. Researchers can now integrate predictive technology into studies, which similarly raise ethical concerns (2).

We cannot afford to ignore opportunities to prevent suicide nor tools that might enhance patient safety. Yet we must honor our obligations to do so ethically reflecting time-honored tenets such as respect for persons, beneficence, non-malfeasance, and justice. The primary question remains, how can we incorporate AI into research and care practices for suicide prevention while minimizing impact on individual liberty? Our recommendations follow.

## Communication

Public perceptions of data privacy are evolving, with increased reluctance to share personal data. Do patients want AI surveilling their healthcare data? We need public discussion among stakeholders, providers, and patients prior to scaling AI into healthcare systems. In this population, relevant stakeholders include those at high risk of and with histories of suicidality, familial survivors of suicide, their caregivers, their support networks, and those who might direct resources or regulation toward suicide prevention. The benefits of these conversations include: (1) the design of more compassionate and transparent systems; (2) the opportunity to lessen stigma around suicide by encouraging its open discussion in community settings.

Education is crucial in translating AI models into practice. Even health numeracy challenges both providers and patients, so the difficulty of understanding AI and its potential implications is non-trivial. Data scientific curricula specific to engaging smart agents will be required, including culturally-sensitive AI educational materials tailored to all levels of health literacy. A strong precedent exists for specific education of the ethical challenges, unique factors, and potential harms to provider well-being in encountering suicide in practice (14). Furthermore, education for surrogates such as parents in pediatric settings will require additional effort. Patients require active engagement to enhance communication in conversations prompted by “our risk algorithm identified you as at-risk.”

## Consent

Healthcare systems are ethically bound to inform both patients and providers (end-users) of AI risk models that these algorithms (1) can be imperfect or wrong; (2) monitor data considered highly sensitive or confidential (e.g., psychiatry notes); (3) might recommend actions that are not immediately apparent; and (4) might prompt intervention without the need to trigger provider action. The need for informed consent in treating suicidal patients has been well-supported throughout the literature and holds true here just as it does throughout healthcare and in other domains impacting individual liberty (14).

Under the most common rubric, imminent risk exists when patients have suicidal intent, plans, and means (14). Providers respond actively to patients' potential for suicide and breach confidentiality if necessary to protect them. Others define imminent risk by foreseeability, or the “reasonable anticipation … of suicide or suicidal behavior in the very near future (p626)” (15). Clinical judgment informed by patient assessment of suicidal potential constitutes a prediction. If AI can produce reliable and valid predictions in this vein, it may be deemed foreseeable risk. Policymakers must balance desirability of action here alongside associated safeguards for privacy and confidentiality.

When foreseeable risk exists, actions promoting patient safety range from safety checks to formal risk assessments. For example, new data from a wearable device may prompt a suicide risk algorithm to alert a provider, the patient, a family member, or the police. These actions all have implications of potential voluntary or involuntary hospitalization and consequences to patient autonomy and privacy.

We recommend the following risk mitigation steps (Table 1): First, when presenting for treatment, during informed consent, patients with decisional capacity should have the right to “opt-out” of AI monitoring even at the expense of increased system complexity to support this right. How and when to best integrate such consent remain open questions. We have an obligation to respect choice and an individual's right to privacy. Second, we need to study and develop consent forms that account for AI's ability to evolve far more quickly than guidelines can be updated. This concern includes improved consent regarding (1) types, breadth, and depth of data collected and (2) potential risks of AI in practice including those outlined here. Moreover, consent may need to expire to prompt reevaluation of AI–analogous to continuing institutional review board review for ongoing studies to ensure benefits continue to exceed harms.

**Table 1:** Recommendations for risk mitigation applying AI for suicide prevention in healthcare settings.

**Domain**	**Recommendation for implementation**	**Recommendation for research**
Consent	Develop informed consent for patients to sign detailing the actions and limitations of AI	Develop consent forms to all literacy levels and test for understanding
	Develop similar consent for providers	Develop patient education materials that detail the purpose of AI and evaluate for understanding
	Provide patients with “opt-out” of AI monitoring
	Provide time limits or expiration to consent
	Re-consent each year with evolving technology
	Have consent documents approved by experts and medical review board
Controls	Adopt standards for suicide monitoring with AI, such as determining what percentage of at-risk individuals will be monitored	Compare provider-informed vs. AI-only model to assess for increased accuracy with feedback
	Form an AI oversight panel with multidisciplinary specialty
	Request provider feedback routinely and update systems accordingly
	Create a system for providers to defer or activate risk monitoring with explanation	
	Log model successes and failures, re-train models
Communication	Conduct focus groups with stakeholders to assess for appropriateness and utility of integrating AI into healthcare	Develop provider materials and elicit feedback for appropriateness
	Provide communication materials for provider use to discuss AI and the monitoring process	

## Controls

Potential benefits of AI-based risk prediction include identifying those at acute risk needing intervention. In large hospital systems, even the highest 1% translates to thousands of individuals monitored. Workflows supporting such alerts do not exist in current practice. Similarly, algorithms can produce aberrant predictions labeling individuals as “high-risk” erroneously due to model mis-specification, data collection glitches, or faults introduced during model or software updates. We have an obligation to evaluate AI tools prospectively throughout their use–not simply for pilot or study trial periods. Further, we must learn from providers before, during, and after implementation of AI tools along with analyzing model successes and failures. To achieve the “learning health system,” implementations need to support feedback loops for providers to correct misclassification from AI and vice-versa (16). To test whether AI yields valid and reliable predictions of suicide risk, prospective evaluation of such AI, e.g., in a pragmatic trial, might directly compare AI predictions with clinician best judgment. Moreover, assessing the additive value of combining both AI predictions and clinical judgment might have important implications for suicide prevention. These opportunities will improve model accuracy and implementation.

As computational models become more sophisticated, they become less easy to interrogate, which has implications for both Communication and Controls. For the latter, this lack of interpretability compounds the potential harms of imperfect models (17). Not only can models miscalculate, this may occur in unexpected or difficult to parse ways. Erroneous predictions threaten provider trust in AI, and could ultimately compromise AI uptake. And the factors leading to errors might be hard to repair. Periodic evaluation of models prospectively will prevent drift in both performance and interpretability.

## A note on applied vs. general artificial intelligence

The vast majority of AI integration in healthcare today exemplifies Applied Artificial Intelligence, which is currently being explored in healthcare systems and the focus of our discussion. Much of the attention in the media in fact focuses on the concept of Artificial General Intelligence (AGI)–a concept closer to truly autonomous systems and agents (18). It is conceivable that autonomous AGI might one day be applied in mental health and suicide risk prediction and similarly that this system might consider and draw its own conclusions about mental health surveillance and intervention. Ethical considerations in light of AGI will require a continual and iterative reassessment of principles that might govern such systems. One might imagine such a system participating in the ethical discussion. We reiterate this possibility remains theoretical and likely far off in the timeline of healthcare information technology but feel it deserves mention given the rapid advancement in machine learning that has already changed the biomedical literature.

In healthcare settings, AI is already being implemented at scale to prevent suicide (19, 20). With appropriate communication, consent, and controls, ethical application of AI can succeed as it has in preventing gun violence in Chicago (21). To approach Zero Suicide, novel methods like AI are needed to identify those at risk who would otherwise be missed. Implementation of such technology demands an ethical framework to ensure benefits outweigh risk and to prioritize the welfare of the patient. The potential partnerships between smart humans and smart agents may shine light on the patients at most risk, wherever they are, rather than hoping those patients walk into the light of a routine or emergent healthcare encounter. In suicide, this last hope often comes too late.

## Author contributions

All authors on the manuscript (LM, EC, and CW) were involved in the conceptualization, writing, and revising of this manuscript.

### Conflict of interest statement

The authors declare that the research was conducted in the absence of any commercial or financial relationships that could be construed as a potential conflict of interest.
